# Rapid, sensitive, type specific PCR detection of the E7 region of human papillomavirus type 16 and 18 from paraffin embedded sections of cervical carcinoma

**DOI:** 10.1186/1750-9378-5-2

**Published:** 2010-01-22

**Authors:** Iana Lesnikova, Marianne Lidang, Steven Hamilton-Dutoit, Jørn Koch

**Affiliations:** 1 Institute of Pathology, Århus University Hospital, Nørrebrogade 44, 8000 Århus C, Denmark; 2 Institute of Pathology, Herlev Hospital, Herlev Ringvej 75 2730 Herlev, Denmark

## Abstract

Human papillomavirus (HPV) infection, and in particularly infection with HPVs 16 and 18, is a central carcinogenic factor in the uterine cervix. We established and optimized a PCR assay for the detection and discrimination of HPV types 16 and 18 in archival formaldehyde fixed and paraffin embedded (FFPE) sections of cervical cancer.

Tissue blocks from 35 cases of in situ or invasive cervical squamous cell carcinoma and surrogate FFPE sections containing the cell lines HeLa and SiHa were tested for HPV 16 and HPV18 by conventional PCR using type specific primers, and for the housekeeping gene β-actin. Using HPV 16 E7 primers, PCR products with the expected length were detected in 18 of 35 of FFPE sections (51%). HPV 18 E7 specific sequences were detected in 3 of 35 FFPE sections (9%).

In our experience, the PCR technique is a robust, simple and sensitive way of type specific detection of HPV16 and HPV18 genes in FFPE tissue. That makes this technique applicable to routine practices of HPV detection.

## Findings

Human papillomavirus (HPV) infection is a central carcinogenic factor in the uterine cervix, and cervix cancer is a common malignancy in women worldwide [1]. Most women with squamous epithelial dysplasia or invasive squamous cell carcinoma of the cervix are infected with high risk HPV types, particularly HPVs 16 and 18 [2,3]. HPV E6 and E7 oncoproteins interfere with the function of the tumor suppressor proteins p53 and pRB, leading to hyperproliferation and genomic instability [2,4,5].

The new vaccination program should confer type specific immunity against HPV 16 and 18, and it is expected to be a valuable tool for reducing the incidence of cervical cancer [6]. To evaluate the efficacy of vaccination, it is useful to determine the HPV types found in routine cervical samples from vaccinated persons.

In the present study we aimed to establish sensitive, specific and simple assays for type specific detection of the presence of the E7 region of HPV 16 and 18 DNA in FFPE tissue, since fresh unfixed tissue is rarely available for routine diagnostics. Methods to this end, such as HPV detection by PCR using the MY09/MY11 and GP5+/GP6+ primer systems have been described, but have often been found to allow only the broad detection of a spectrum of HPVs [7,8]. A number of PCR based detection methods of high risk HPV in unfixed sections have been reported, but only a few reports on PCR detection of HPV in FFPE cervical cancer sections exist [9]. Thus we desired to create a more robust type specific detection - so robust that it would even work on FFPE material.

In general the sensitivity of PCR-based assays is lower in FFPE specimens compared with fresh, unfixed tissues as a result of degradation and fragmentation of the target DNA [10-12]. Typically, DNA sequences extracted from FFPE tissues are found in fragments with a length of 200 bp or less [10,12,13].

Tissue blocks from 35 cases of loop excision or surgical excision of carcinoma in situ or of invasive cervical squamous carcinoma were randomly selected in the pathology database "Snomed" and obtained from the archives of the Institute of Pathology, Aarhus University Hospital. All were routine diagnostic surgical specimens that had been fixed in buffered formalin and embedded in paraffin. Specific fixation times were not known for individual specimens, but typically varied between 18 and 24 hours. Cases were classified on haematoxylin and eosin stained sections by a pathologist experienced in evaluation of gynaecological pathology according to standard World Health Organization diagnostic criteria

We used the following cell lines as controls in our study: HeLa (contains about 50 copies of HPV 18 according to the supplier), SiHa (contains 2-5 copies of HPV 16 according to the supplier) and fetal kidney cell line 293-EBNA (does not contain HPV) [14]. Cell lines cells were fixed in 4% buffered formaldehyde pH-7 (VWR international Geldenaaksenbaan 464, B-3001 Leuven Belgium), centrifuged, and the cell pellets were embedded in paraffin in order to simulate the routine fixation procedure.

DNA isolation from unfixed Hela, SiHa and 293-EBNA cell lines was performed using a QIAmp DNA purification kit (QIAGEN, http://www.qiagen.com) according to the manufacturer's protocol.

DNA isolation from FFPE tissue was performed the following way: three 15 μm paraffin sections were cut from each FFPE specimen and placed in an eppendorf tube. In order to prevent cross-contamination, a new microtome blade was used for each specimen. Sections were deparaffinized by incubation for 2 × 20 min at 60°C in Tissue-Clear (Sakura Finetek Europe, 2382 AT, Zoeterwoude, NL), then placed in 99% ethanol for 2 × 20 min at 60°C and air-dried. They were subsequently resuspended in 300 μl of digestion buffer (50 mM Tris-HCl [pH 7.5], 10 mM EDTA, 0.5% sodium dodecyl sulfate, 50 mM NaCl, and 1.5 mg/ml proteinase K [Roche, Basel, Switzerland]) and incubated at 56°C under rocking conditions for 24 hours until the tissue was completely digested. The proteinase K was inactivated by incubating the samples for 20 min at 95°C. Phenol/chloroform/isoamyl alcohol (25:24:1; v/v) was added to each tube at a volume equal to that of the digestion mixture (300 μl). Tubes were then vortexed for 10-15 sec and centrifuged at 14,000 rpm for 5 min. The supernatant was transferred to new tubes and mixed with 1/10 volume of 3 M sodium acetate and 2.5 volume of 99% ice-cold ethanol, incubated at -20°C for 30 min, then centrifuged at 14,000 rpm for 5 min, and the supernatant was discarded. Pellets were washed with 1 ml of 70% ethanol, centrifuged for 5 min at 14,000 rpm, after which the ethanol was removed and the specimen air-dried. Dry pellets were then resuspended in water. The integrity and purity of the DNA extraction and the concentration of the extracted DNA was assessed by spectrophotometry.

DNA extracted from unfixed cells and from FFPE sections was used as template for PCR reactions. Amplification reactions were performed with an AmpliTaq Gold PCR kit (Applied Biosystems, CA). The reaction mixture with a total volume of 25 μL contained 1 × AmpliTaq Gold buffer, 5.5 mM MgCl_2_, 100 nM of each dNTP, 300 nM of each primers, and 0.6 U AmpliTaq Gold polymerase. The DNA template concentrations varied from 5 ng to 500 ng per reaction. The primers used for the β-actin detection are listed in table 1. The amplification was performed in two steps where 2.5 μL of the PCR product from step 1 was used as a template in step 2. The PCR cycling conditions were 95°C 12 min, (60°C 1 min, 72°C 1 min, 95°C 30 sec) × 40 cycles, 72°C 1 min. Ten μl of PCR product was run in 1.5% agarose gel at 80 V for 90 min and stained with ethidium bromide, to demonstrate the expected PCR product length of 99 bp.

**Table 1:** PCR primers used

Amplified region	Primer	Sequences	Melting temperature	Amplimer length
HPV16 E7	Pr. 591-620	5'ATA TAT GTT AGA TTT GCA ACC AGA GAC AAC 3'	55.9°C	196 bp
		
	Pr. 786-762	5'GTC TAC GTG TGT GCT TTG TAC GCA C 3'	54.2°C	

HPV18 E7	Pr. 533-553	5'CCG AGC ACG ACA GGA GAG GCT 3'	60.3°C	172 bp
		
	Pr. 705-682	5' TCG TTT TCT TCC TCT GAG TCG CTT 3'	57.5°C	

β-actin	Pr. 6999-7018	5'CCACACTGTGCCCATCTACG3'	53.6°C	99 bp
		
	Pr. 7097-7072°C	5'AGGATCTTCATGAGGTAGTCAGTCAG3'	54.0°C	

DNA extracted from both unfixed cells and FFPE sections was used as template for type specific HPV 16 and HPV 18 detection. Amplification reactions were performed with the FastStart High Fidelity PCR system (Roche). Primers are listed in table 1, and gave an expected PCR product length of 196 bp and 172 bp respectively for HPV 16 and HPV 18. Reaction mixtures with a total volume of 50 μl contained 1 × reaction buffer without Mg^2+^, 3.0 mM MgCl_2_, 200 μM of each dNTP, 0.4 μM of each primers, and 2.5 U FastStart High Fidelity Taq polymerase. DNA template concentrations varied from 5 ng to 500 ng per reaction. The amplification was performed in two steps: PCR cycling 95°C 5 min, (95°C 30 sec, ramp from 57°C to 48°C, 1°C per cycle, 72°C 1 min) 10 cycles, (95°C 30 sec, 48°C 30 sec, 72°C 1 min) × 25, 72°C 5 min. Ten μl of PCR product were run in 1.5% agarose gel at 80 V for 90 min and stained with ethidium bromide.

**Figure 1:**
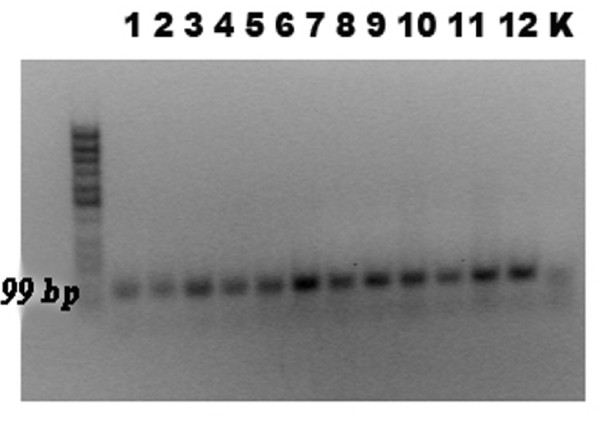
**Analysis of the β-actin housekeeping gene in FFPE sections of cervical cancer (lanes 1-12)**. Following PCR, products were electrophoresed in 1.5% agarose gel (amplicon length 99 bp).

To evaluate the quality of the DNA extracted from the study specimens for amplification by PCR, all samples were tested for the housekeeping gene β-actin using PCR. β-actin DNA was detected in 32 of 35 (91%) FFPE, in situ or invasive cervical squamous carcinoma samples whereas β-actin was detected in all cell line samples (table 2; figure 1). Negative controls were consistently negative for β-actin.

**Figure 2:**
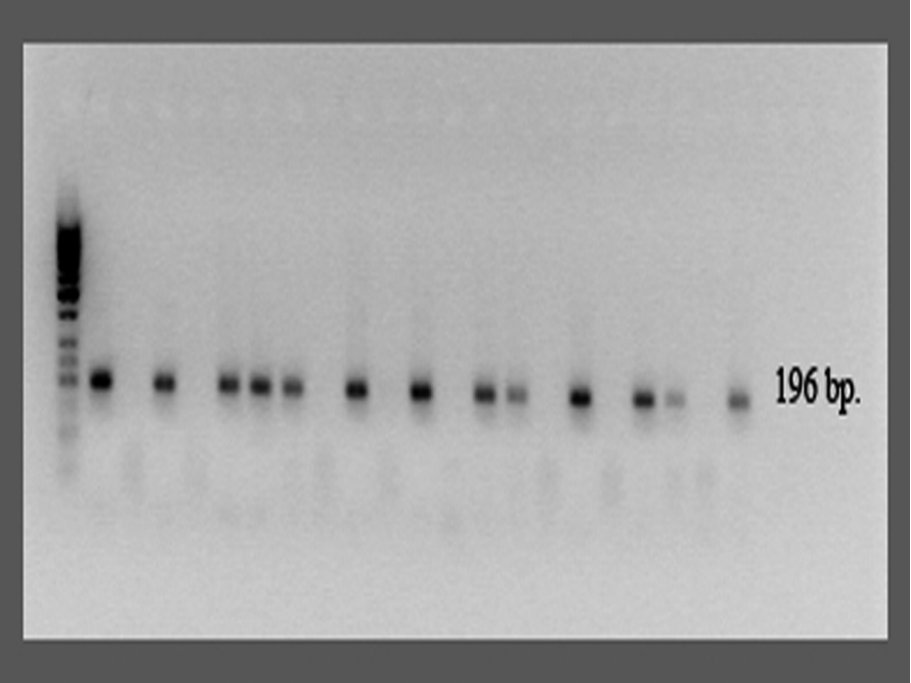
**Analysis of HPV 16 E7 in FFPE material**. Following PCR, products were electrophoresed in 1.5% agarose gel (amplicon length 196 bp). Lane 1 represents positive control DNA (from HPV-16 positive SiHa cell line); lane 2 is a negative control (no template); a lane 3-21 represents DNA from FFPE sections of cervical cancer.

**Figure 3:**
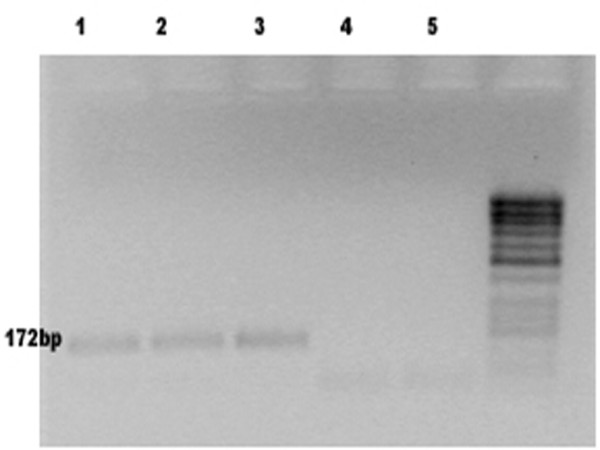
**Analysis of HPV 18 E7 in FFPE material**. Following PCR, products were electrophoresed in 1.5% agarose gel (amplicon length 172 bp). Lane 1 and 2: DNA from FFPE study specimens; lane 3 is a positive control (DNA from HPV 18-positive HeLa cell line); lane 4 is a negative control (DNA from SiHa cell line); lane 5 is a negative control (no template).

All samples and controls were tested for HPV 16 E7 and HPV 18 E7 using specific primers within the E7 regions of the two genomes. Primers were designed using the "Vector NTI" software. The HPV16, HPV18 and β-actin sequences were obtained from the NCBI database. Results are shown in table 2. No amplification products were seen in the 293-EBNA (HPV-negative) cell line used as a negative control. The expected PCR product of 196 bp was detected using HPV 16 E7 primers in both unfixed and FFPE SiHa cell lines. In both unfixed and FFPE HeLa cell lines, a PCR product of the expected 172 bp length was detected using primers complementary to the HPV 18 E7 region. We detected no evidence of cross reactions between the HPV 16 E7 and the HPV 18 E7 regions. Using HPV 16 E7 primers, PCR products with the expected length of 196 bp were detected in 18 of 35 of FFPE sections (51%) (figure 2), whereas HPV 18 E7 specific sequences were detected in 3 of 35 FFPE sections (9%) (figure 3).

**Table 2:** Results of type specific PCR detection of HPV 16 E7, HPV 18 E7 and β-actin in FFPE sections of cervical cancer.

Case number	HPV16 E7	HPV18 E7	β-actin
HeLa unfixed	-	+	+

HeLa FFPE	-	+	+

SiHa unfixed	+	-	+

SiHa FFPE	+	-	+

EBNA unfixed	-	-	+

1	+	-	-

2	-	-	+

3	+	-	+

4	+	-	+

5	+	-	+

6	+	-	+

7	-	-	+

8	+	+	+

9	-	-	+

10	+	-	+

11	-	-	+

12	+	-	+

13	+	-	+

14	-	+	+

15	+	-	+

16	-	-	+

17	+	-	+

18	+	-	+

19	-	-	-

20	+	-	-

21	-	-	+

22	-	-	+

23	-	-	+

24	-	-	+

25	-	-	+

26	-	+	+

27	-	-	+

27	-	-	+

29	+	-	+

30	-	-	+

31	+	-	+

32	-	-	+

33	+	-	+

34	-	-	+

35	-	-	+

Cell lines SiHa (containing HPV 16), HeLa (containing HPV 18) and 293-EBNA (HPV negative) were used as controls

We designed and optimized our PCR assay for the detection of HPV in potentially damaged DNA templates. In accordance with previous studies showing that PCR-based detection assays in FFPE sections work best when the amplicon size is up to 200 bp, amplicon sizes from 99 bp to 196 bp were used [11,15]. To validate the suitability of each study block, DNA extracted from FFPE sections was analyzed using PCR for the single copy housekeeping gene β-actin. This gene could not be detected in 3 of 35 specimens (9%) although in 2 of these cases, specific HPV sequences could be amplified. This suggests that our HPV targets were easier to detect than the β-actin gene. This may reflect that the HPV genes are present in the tissues in multiple copies per cell, as compared with the single copy β-actin gene.

We chose to place the amplified region of HPV DNA in the E7 regions of HPV DNA, as the expression of the HPV 16 and HPV 18 oncogenes E6 and E7 shows a strong positive correlation with the development of invasive cervical carcinoma [4,5]. In order to improve type specific detection of HPVs 16 and 18, we chose PCR primers from gene regions with maximum sequence difference between the two types of HPV. Our method proved sensitive and specific for the HPV 16 and 18 types, as we detected the expected HPV positivity in both unfixed and surrogate FFPE tissues containing HeLa and SiHa cell lines with no cross reactions between the two types of HPV.

Using HPV 16 E7 primers, PCR products were detected in 51% of sections, whereas HPV 18 E7 specific sequences were detected in 9% of sections. These frequencies correspond with Zur-Hausen et al. who reported that HPV 16 was found in approximately 50% of all cervical cancer and HPV 18 in close to 20% [2,16].

We conclude that the yield and quality of the DNA extracted from the archival FFPE sections of cervical carcinoma was sufficient for PCR assays. Using our optimized PCR protocol, we were able to specifically detect the HPV types 16 and 18 in routine pathology archive specimens with a simple, reproducible and cheap method.

The assay could be applied for post-vaccination typing of HPV types where a universal PCR for HPV identifies any HPV infection, whereas this assay identifies the subpopulation of infections caused by HPV 16 and 18.

## List of abbreviations

DNA: Deoxyribonucleic acid; FFPE: Formalin fixed paraffin embedded; HPV: Human papilloma virus; PCR: Polymerase chain reaction.

## Competing interests

The authors declare that they have no competing interests.

## Authors' contributions

IL obtained the diagnostic material from the archive of the Institute of Pathology, carried out molecular tests and drafted the manuscript. ML carried out pathological examination. SHD carried out pathological examination, participated in the coordination of the study and contributed to the preparation of manuscript. JK participated in the coordination of the study, coordination of the molecular tests, and the final preparation of manuscript. All authors read and approved the final manuscript.
